# Supplementary figures and images for: Machine Learning Reveals Time-Varying Microbial Predictors with Complex Effects on Glucose Regulation
Source: mSystems. 2021 Feb 16;6(1):e01191-20. doi: 10.1128/mSystems.01191-20 (PMC8573957; doi:10.1128/mSystems.01191-20)

A

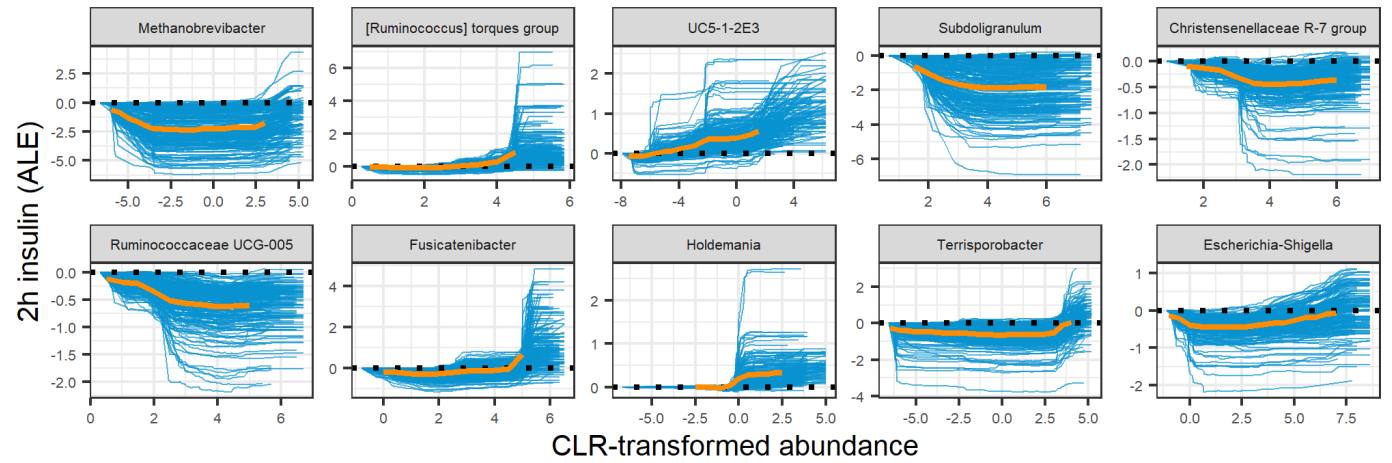

B

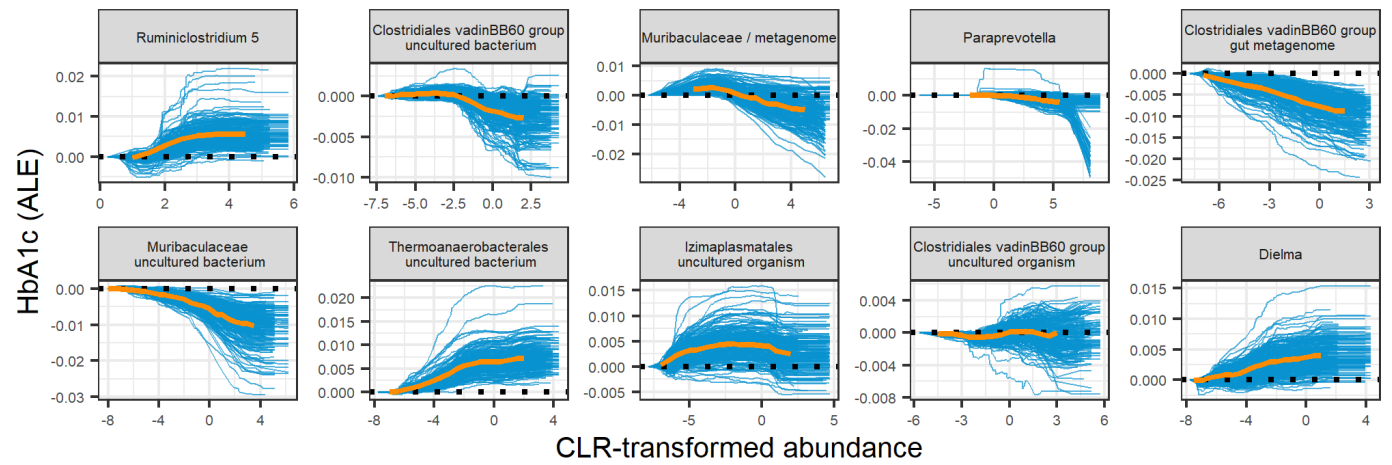

C

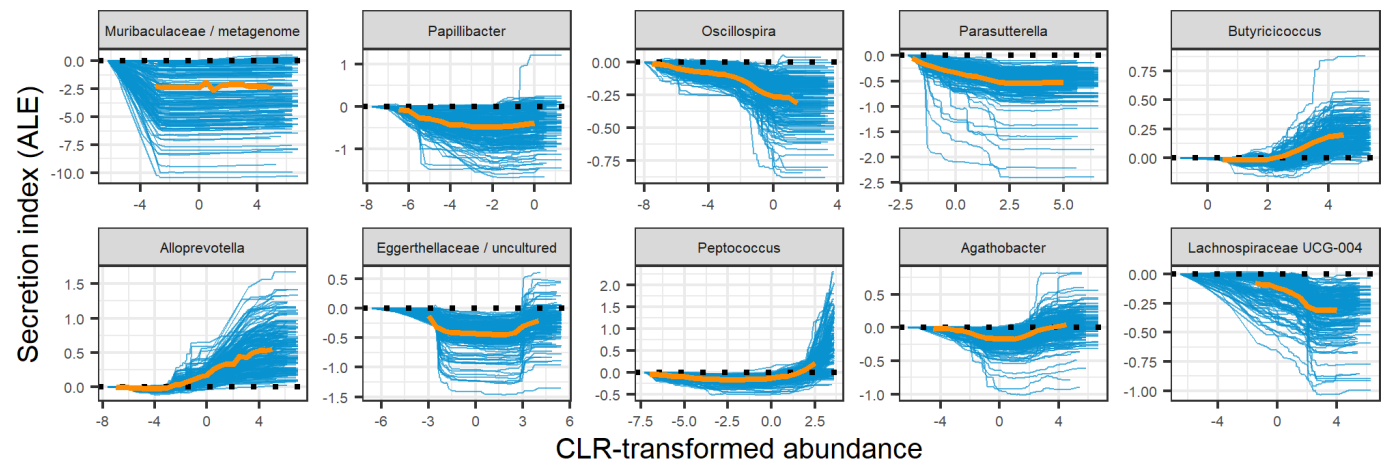

Supplement: FIG S1 [file msystems.01191-20-sf001.pdf]

**A**

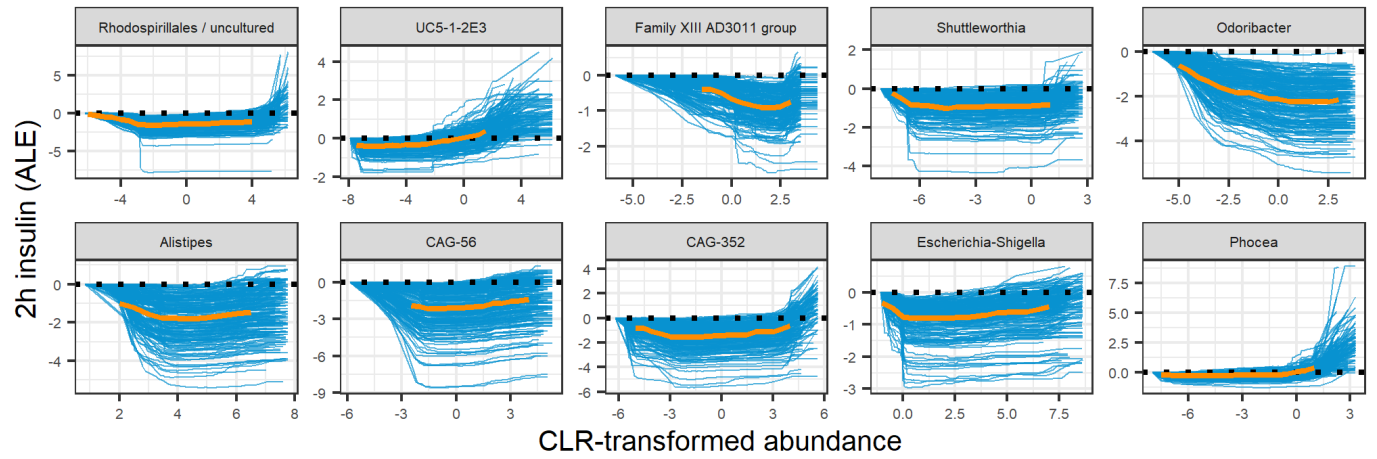

**B**

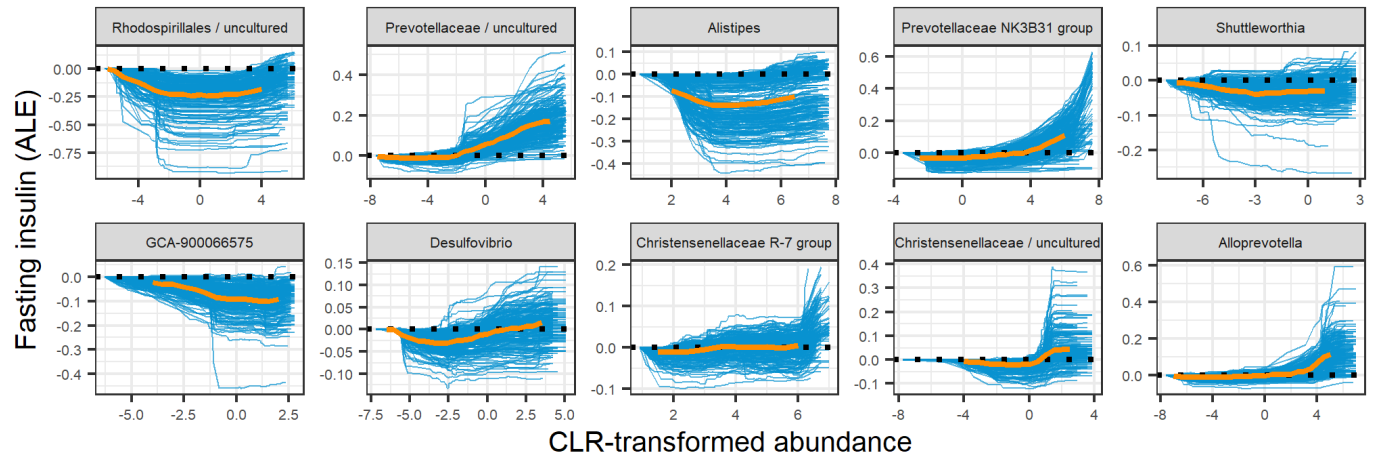

**C**

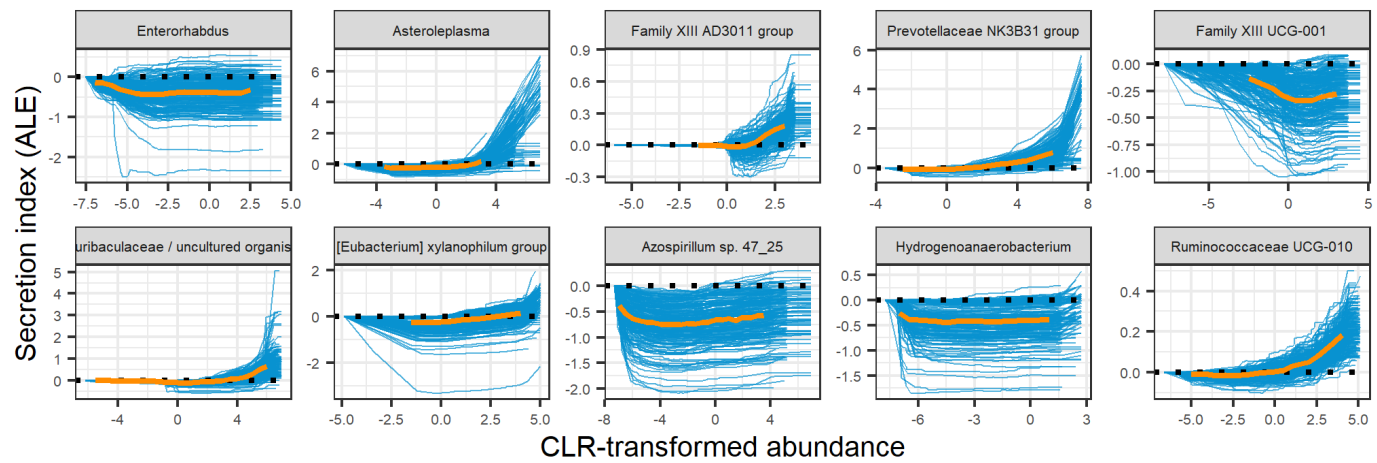

Supplement: FIG S2 [file msystems.01191-20-sf002.pdf]

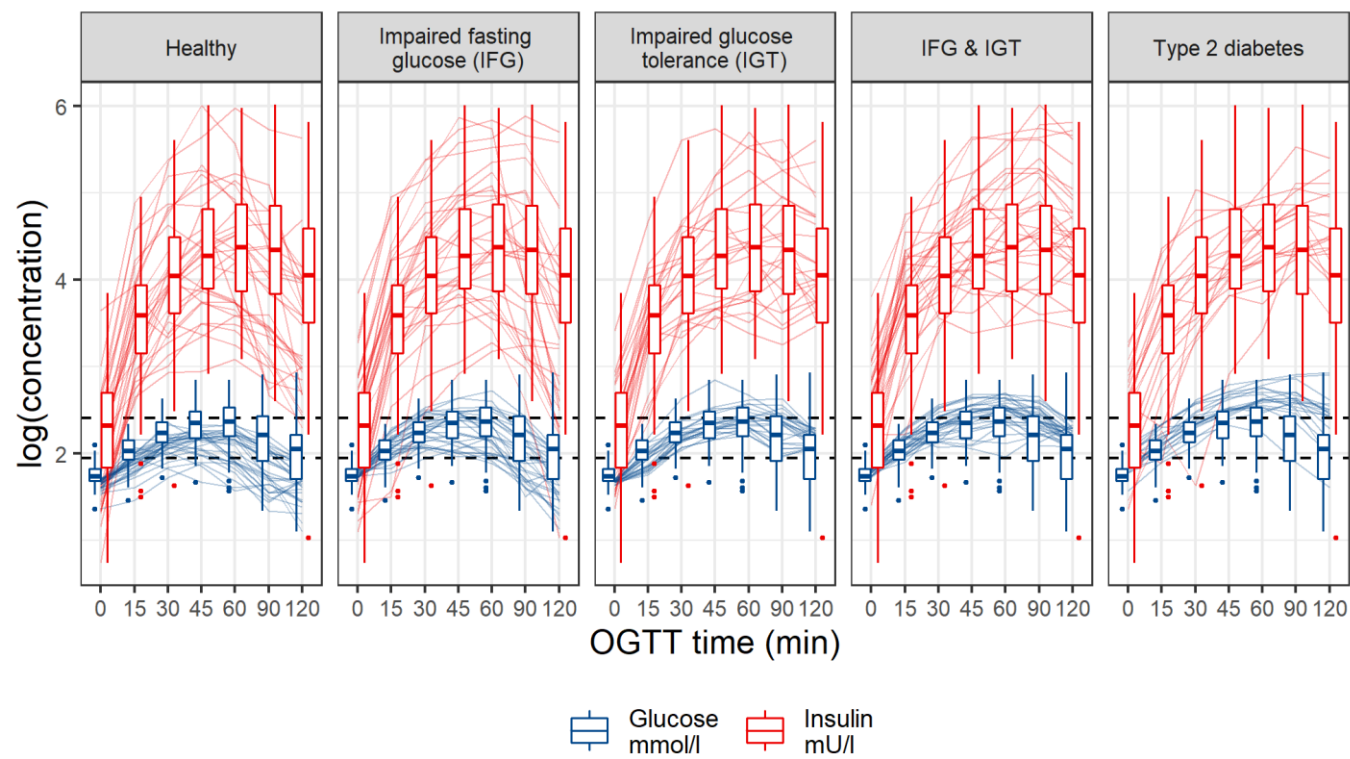

Supplement: FIG S3 [file msystems.01191-20-sf003.pdf]
