# Supplementary material for: Machine Learning Reveals Time-Varying Microbial Predictors with Complex Effects on Glucose Regulation
Source: mSystems. 2021 Feb 16;6(1):e01191-20. doi: 10.1128/mSystems.01191-20 (PMC8573957; doi:10.1128/mSystems.01191-20)
Supplement: TABLE S1 [file msystems.01191-20-st001.docx]

|  | **Baseline**  Mean (sd) | **18-months from baseline**  Mean (sd) | **48-months from baseline**  Mean (sd) |
| --- | --- | --- | --- |
| Age | 62.0 (5.38) | 63.6 (5.40) | 66.1 (5.36) |
| BMI | 27.8 (3.56) | 27.6 (3.63) | 27.7 (3.79) |
| HbA1c (%) | 5.6 (0.29) | 5.6 (0.27) | 5.7 (0.28) |
| Fasting glucose (mmol/l) | 5.8 (0.49) | 5.8 (0.53) | 6.0 (0.52) |
| 2h glucose (mmol/l) | 6.0 (1.99) | 5.9 (1.63) | 6.4 (1.92) |
| Fasting insulin (mU/l) | 9.5 (6.19) | 9.9 (7.12) | 10.1 (6.29) |
| 2h insulin (mU/l) | 47.8 (47.06) | 49.2 (45.29) | 55.6 (52.58) |
| Secretion index | 34.0 (20.24) | 35.6 (21.96) | 35 (20.39) |
| Matsuda index | 4.8 (3.01) | 4.7 (3.17) | 4.4 (2.95) |
| Disposition index | 125.7 (57.28) | 127.5 (67.15) | 120.9 (63.08) |
| History of elevated blood glucose | 237 (39%) |  |  |
| Diabetes in family | 222 (37%) |  |  |
