# Supplementary material for: Machine Learning Reveals Time-Varying Microbial Predictors with Complex Effects on Glucose Regulation
Source: mSystems. 2021 Feb 16;6(1):e01191-20. doi: 10.1128/mSystems.01191-20 (PMC8573957; doi:10.1128/mSystems.01191-20)
Supplement: TABLE S2 [file msystems.01191-20-st002.docx]

| **Trait** | **Phylum** | **Family** | **Genus** | **Average importance score** |
| --- | --- | --- | --- | --- |
| 2h insulin | Euryarchaeota | Methanobacteriaceae | Methanobrevibacter | 1.64* |
|  | Firmicutes | Lachnospiraceae | [Ruminococcus] torques group | 1.46* |
|  | Firmicutes | Lachnospiraceae | UC5-1-2E3 | 1.38* |
|  | Firmicutes | Ruminococcaceae | Subdoligranulum | 1.33* |
|  | Firmicutes | Christensenellaceae | Christensenellaceae R-7 group | 1.24* |
|  | Firmicutes | Ruminococcaceae | Ruminococcaceae UCG-005 | 1.15 |
|  | Firmicutes | Lachnospiraceae | Fusicatenibacter | 1.12 |
|  | Firmicutes | Erysipelotrichaceae | Holdemania | 1.11 |
|  | Firmicutes | Peptostreptococcaceae | Terrisporobacter | 1.04 |
|  | Proteobacteria | Enterobacteriaceae | Escherichia-Shigella | 1.03 |
| HbA1c | Firmicutes | Ruminococcaceae | Ruminiclostridium 5 | 1.11* |
|  | Firmicutes | Clostridiales vadinBB60 group | uncultured bacterium | 1.07* |
|  | Bacteroidetes | Muribaculaceae | metagenome | 1.04* |
|  | Bacteroidetes | Prevotellaceae | Paraprevotella | 1.02* |
|  | Firmicutes | Clostridiales vadinBB60 group | gut metagenome | 0.99* |
|  | Bacteroidetes | Muribaculaceae | uncultured bacterium | 0.84 |
|  | Firmicutes | Clostridiales vadinBB60 group | Uncultured Thermoanaerobacterales bacterium | 0.82 |
|  | Tenericutes | uncultured organism | uncultured organism | 0.81 |
|  | Firmicutes | Clostridiales vadinBB60 group | uncultured organism | 0.78 |
|  | Firmicutes | Erysipelotrichaceae | Dielma | 0.78 |
| Secretion index | Bacteroidetes | Muribaculaceae | metagenome | 0.95* |
|  | Firmicutes | Ruminococcaceae | Papillibacter | 0.79* |
|  | Firmicutes | Ruminococcaceae | Oscillospira | 0.76* |
|  | Proteobacteria | Burkholderiaceae | Parasutterella | 0.67 |
|  | Firmicutes | Ruminococcaceae | Butyricicoccus | 0.67 |
|  | Bacteroidetes | Prevotellaceae | Alloprevotella | 0.65 |
|  | Actinobacteria | Eggerthellaceae | uncultured | 0.64 |
|  | Firmicutes | Peptococcaceae | Peptococcus | 0.63 |
|  | Firmicutes | Lachnospiraceae | Agathobacter | 0.63 |
|  | Firmicutes | Lachnospiraceae | Lachnospiraceae UCG-004 | 0.62 |
