# Supplementary material for: Machine Learning Reveals Time-Varying Microbial Predictors with Complex Effects on Glucose Regulation
Source: mSystems. 2021 Feb 16;6(1):e01191-20. doi: 10.1128/mSystems.01191-20 (PMC8573957; doi:10.1128/mSystems.01191-20)
Supplement: TABLE S3 [file msystems.01191-20-st003.docx]

| **Trait** | **phylum** | **family** | **genus** | **Average importance score** | |
| --- | --- | --- | --- | --- | --- |
| 2h insulin | Proteobacteria | Rhodospirillales (uncultured) | gut metagenome | 1.79* |  |
|  | Firmicutes | Lachnospiraceae | UC5-1-2E3 | 1.73* |  |
|  | Firmicutes | Family XIII | Family XIII AD3011 group | 1.56* |  |
|  | Firmicutes | Lachnospiraceae | Shuttleworthia | 1.52* |  |
|  | Bacteroidetes | Marinifilaceae | Odoribacter | 1.50* |  |
|  | Bacteroidetes | Rikenellaceae | Alistipes | 1.46 |  |
|  | Firmicutes | Lachnospiraceae | CAG-56 | 1.44 |  |
|  | Firmicutes | Ruminococcaceae | CAG-352 | 1.44 |  |
|  | Proteobacteria | Enterobacteriaceae | Escherichia-Shigella | 1.42 |  |
|  | Firmicutes | Ruminococcaceae | Phocea | 1.40 |  |
| Fasting insulin | Proteobacteria | Rhodospirillales (uncultured) | gut metagenome | 0.74* |  |
|  | Bacteroidetes | Prevotellaceae | uncultured | 0.65* |  |
|  | Bacteroidetes | Rikenellaceae | Alistipes | 0.62* |  |
|  | Bacteroidetes | Prevotellaceae | Prevotellaceae NK3B31 group | 0.52 |  |
|  | Firmicutes | Lachnospiraceae | Shuttleworthia | 0.52 |  |
|  | Firmicutes | Lachnospiraceae | GCA-900066575 | 0.51 |  |
|  | Proteobacteria | Desulfovibrionaceae | Desulfovibrio | 0.51 |  |
|  | Firmicutes | Christensenellaceae | Christensenellaceae R-7 group | 0.51 |  |
|  | Firmicutes | Christensenellaceae | uncultured | 0.49 |  |
|  | Bacteroidetes | Prevotellaceae | Alloprevotella | 0.49 |  |
| Secretion index | Actinobacteria | Eggerthellaceae | Enterorhabdus | 0.56* |  |
|  | Firmicutes | Erysipelotrichaceae | Asteroleplasma | 0.45* |  |
|  | Firmicutes | Family XIII | Family XIII AD3011 group | 0.42* |  |
|  | Bacteroidetes | Prevotellaceae | Prevotellaceae NK3B31 group | 0.40 |  |
|  | Firmicutes | Family XIII | Family XIII UCG-001 | 0.40 |  |
|  | Bacteroidetes | Muribaculaceae | uncultured organism | 0.40 |  |
|  | Firmicutes | Lachnospiraceae | [Eubacterium] xylanophilum group | 0.39 |  |
|  | Proteobacteria | uncultured | Azospirillum sp. 47_25 | 0.38 |  |
|  | Firmicutes | Ruminococcaceae | Hydrogenoanaerobacterium | 0.37 |  |
|  | Firmicutes | Ruminococcaceae | Ruminococcaceae UCG-010 | 0.37 |  |
